# Lyme disease presenting as complete heart block in a young man: Case report and review of pathogenesis

**DOI:** 10.1016/j.idcr.2023.e01799

**Published:** 2023-05-12

**Authors:** Maria Chiara Carnazzo, Celine Scholin, FNU Shweta, Andrew D. Calvin

**Affiliations:** a Post-graduate School of Emergency Medicine, Faculty of Medicine and Surgery, University of Modena and Reggio Emilia, Modena, MO 41125, Italy; b Medical College of Wisconsin - Central Wisconsin, Wausau, WI 54401, United States; c Department of Infectious Disease, Mayo Clinic Health System, Eau Claire, WI 54703, United States; d Department of Cardiovascular Medicine, Mayo Clinic Health System, Eau Claire, WI 54703, United States

**Keywords:** Lyme disease, Lyme carditis, Cardiotropism, Host factors, AV block, Pacemaker

## Abstract

Lyme carditis is a serious complication of Lyme disease, the most common vector-borne infection in both the United States and Northern Europe. It is a rare manifestation of Lyme disease that primarily affects young adults with a marked 3:1 male-to-female predominance. The presentation of Lyme carditis is heterogenous and often non-specific, although the most common clinical manifestation is AV block, which can be acute in onset and can rapidly progress to complete heart block. We discuss the case of a young adult male with complete heart block as a complication of Lyme infection, presenting with two episodes of syncope without prodromal symptoms months after tick bites. There are several pathogen, host and environmental factors that can play an important role in the epidemiology and pathogenesis of this serious condition that is reversible if treated in a timely manner. It is important for clinicians to be familiar with the presentation and treatment of this infection that is now being observed in a wider geographic distribution so as to avoid serious long-term complications and unnecessary permanent pacemaking implantation.

## Introduction

Lyme disease is the most common vector-borne infection in both the United States and Northern Europe. In 2018, the CDC reported over 33,000 Lyme disease cases in the US [1] and the incidence is increasing, largely driven by climate change and reforestation [2], [3]. Lyme infections are caused by the spirochete bacteria *Borrelia burgdorferi* sensu lato, a complex of 20 different genospecies, transmitted through the bite of the Ixodes tick [2], [4]. *B. burgdorferi sensu stricto* is the most prevalent in the United States, while *B. garinii* and *B. afzelii* are more common in Europe [4]. *Borrelia burgdorferi* was first discovered in 1982 in the United States. It was later recognized that the strains of *B. burgdorferi* present in Europe are more heterogenous than those in North America [2], [4]. Just as the etiologic agents and related tick vectors of Lyme disease vary between Europe and the US, the disease’s clinical manifestations vary as well [4]. Lyme disease can evolve through 3 stages that characteristically affect different organ systems at different times. Weeks to months after the initial tick bite, spirochetes spread to various organs, including the heart or joints [2], [5]. Here we describe a case of Lyme carditis, a serious cardiac sequela of Lyme infection.

## Case presentation

**Fig. 1:**
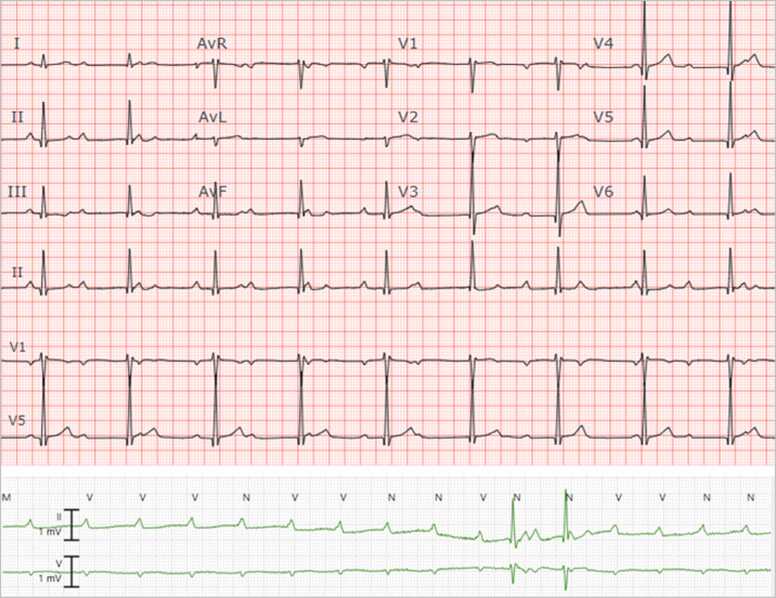
Initial ECG showing normal sinus rhythm with complete heart block with a narrow QRS complex (top) and a rhythm strip showing complete heart block with many non-conducted P-waves and prolonged ventricular pauses.

**Fig. 2:**
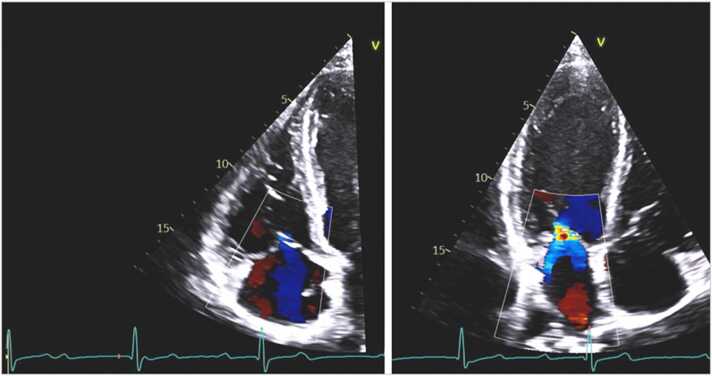
Echocardiography from apical 4-chamber views showing tricuspid regurgitation (left) and mitral regurgitation (right) during diastole. Red marker on the ECG denotes where in the cardiac cycle the image was taken.

**Fig. 3:**
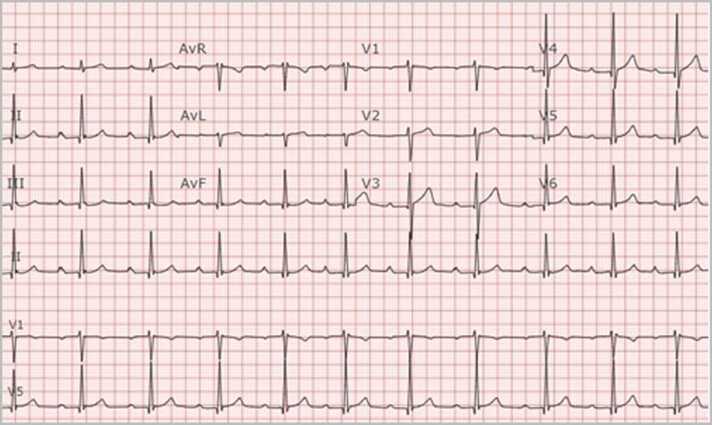
Follow up ECG showing normal sinus rhythm with first degree AV block and a PR interval of 280 ms.

**Fig. 4:**
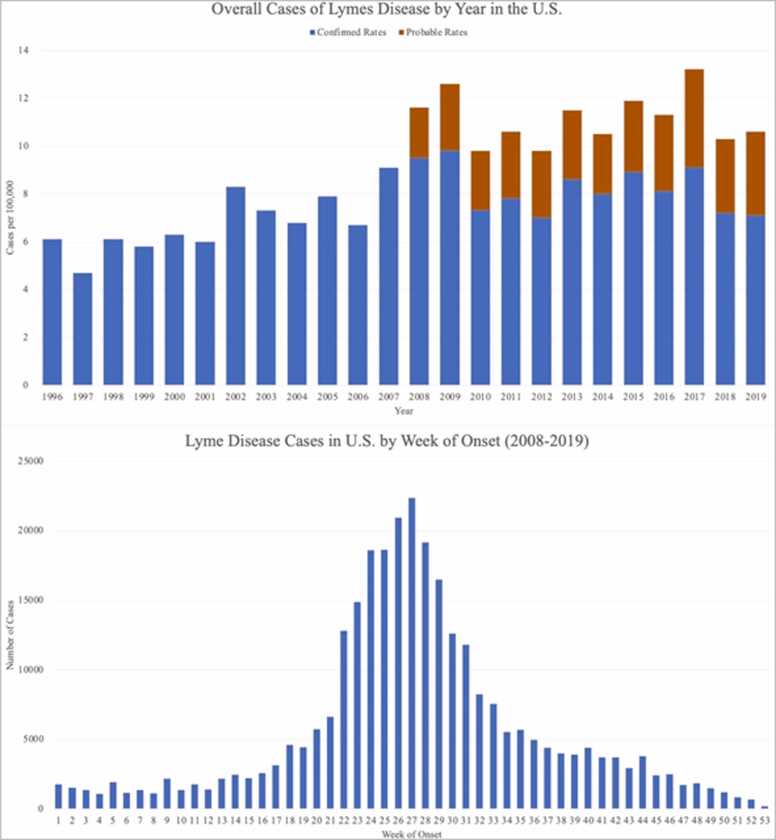
Bar graph showing the increasing incidence of Lyme disease in the United States between the years 1996 and 2019 and the seasonal patterns of Lyme disease infection in the U.S. by week of onset, between the years 2008 and 2019, using CDC data [1]. The patient’s tick bite likely occurred between weeks 14–22 (April-May) and Lyme disease presentation occurred between weeks 27–30 (July).

In July 2022, a 37-year-old previously healthy male who lived and worked in northwestern Wisconsin, presented to the emergency department with two episodes of syncope without prodromal symptoms in the past 5 days. During these days, he had increasingly frequent episodes of dizziness and near-syncope. He also reported fever, chills, muscle aches, and bilateral lower extremity erythematous rash that had developed 3 weeks earlier that had resolved by the time of presentation. The patient reported removing several ticks from his left thigh and groin in April and May of 2022. SARS-CoV-2 tests were negative for active infection and he had no history of recent travel. An initial electrocardiogram (ECG) revealed complete heart block with a heart rate of 55 bpm (Fig. 1), so a temporary pacemaker was urgently inserted. Laboratory testing was positive for *B. burgdorferi* IgM and IgG antibodies on enzyme-linked immunosorbent assay (ELISA) screen and confirmatory western blot. Based on these data, Lyme carditis was diagnosed and he was started on intravenous ceftriaxone. An echocardiogram showed diastolic tricuspid and mitral regurgitation (Fig. 2). After 9 days the complete heart block resolved. Follow up ECG on day 10 showed first degree atrioventricular (AV) node block with a PR interval of 280 ms and a heart rate of 64 bpm (Fig. 3). The patient was then transitioned to oral doxycycline 100 mg PO BID to complete total 4-week course of antimicrobials and was discharged Fig. 4.

## Discussion

Lyme carditis is an uncommon manifestation of Lyme disease that primarily affects males between 20 and 40 years old, consistent with our case, and women 25–29 years old [6], [7]. Cardiac involvement is a rare complication of the early dissemination stage of Lyme disease; it is estimated that 4–10% of patients in the United States with untreated Lyme disease develop carditis and it may be isolated or accompanied by cutaneous (erythema migrans; EM), joint (arthritis), or neurologic (neuroborreliosis) manifestations [2], [4], [5], [8]. Some estimates show the prevalence of asymptomatic carditis as high as 30%; while in other cases symptoms of Lyme carditis are heterogenous and non-specific, including lightheadedness, syncope, dyspnea, palpitations, and chest pain [5]. Preceding history of flu-like symptoms, EM, and travel usually occur 1–2 months prior to cardiac syndrome presentation, contributing to lack of recall in several cases [5].

The most common finding in Lyme carditis is AV block with symptoms such as light-headedness, syncope or near-syncope, shortness of breath, palpitations, and chest pain [5], [9]. Heart block in Lyme carditis can be acute onset and rapidly fluctuating [2], [5], [8], [9]. In up to 90% of cases, Lyme carditis presents as AV block, typically intermittent and shifting from first to third-degree in severity [2], [5], [9], [10]. Among cases of Lyme carditis with high-grade (second-degree type 2 or third-degree) AV block, almost half progressed to complete heart block and 20% included second-degree AV block [5], [10], [11]. Progression of first-degree AV block to complete AV block appears most likely when the PR interval exceeds 300 ms [11], [12]. Other conduction abnormalities of Lyme carditis include left and right bundle branch blocks, diffuse ST segment depression with prominence in the anterolateral leads, and T wave flattening or inversion, typically in the inferolateral leads [13]. Additionally, sick sinus syndrome, atrial fibrillation, isolated tachycardia-bradycardia syndrome, supraventricular and ventricular tachycardias, ventricular fibrillation, pericarditis, myocarditis, endocarditis, pericardial effusion, small vessel vasculitis, cardiomegaly and sudden cardiac death have been reported related to Lyme carditis [2], [5], [9], [13], [14], [15].

## Diagnosis

Prompt recognition of Lyme carditis is important to avoid life threatening complications from the disease and unnecessary treatment such as permanent pacing [11]. Although, appropriately diagnosing Lyme carditis is challenging, requiring confirmation of the association between a patient’s historical, clinical, and laboratory data [5].

Patients presenting with AV block should be asked about possible tick exposure, history of erythema migrans rash, recent travel to a high-incidence Lyme disease area and other constitutional symptoms of Lyme disease like fever, fatigue, malaise, chills, muscle and joint pain [7], [16]. Clinicians should be familiar with the prevalence of Lyme disease in their geographical location. A primarily clinical strategy for diagnosis of Lyme carditis in the setting of AV block can lead to failure to recognize Lyme disease, especially as the geographical distribution of Lyme disease changes related to climate change and northern migration of Ixodes ticks and host animals [2], [3], [16]. Moreover, Lyme carditis can be difficult to recognize in cases where classic signs of Lyme disease are not obvious upon patient presentation and EM rash or tick bite difficult to recall [5], [16]. To address these challenges with the timely diagnosis of Lyme carditis, the Suspicious Index in Lyme Carditis (SILC) has been proposed [14], [17]. This risk score emphasizes important demographic and clinical parameters suggestive of Lyme disease, and patients are classified based on these criteria as low, intermediate, or high risk for the presence of Lyme carditis [14], [17].

Laboratory tests are helpful to support the diagnosis, even if not required in patients with potential tick exposure in a Lyme disease endemic area with history of one or more skin lesions compatible with EM [18]. During the early disseminated phase, most patients present with IgM and IgG seropositivity against *B. burgdorferi* [8]. The most frequent assays used are ELISA and Western blotting [4]. In addition, 12-lead ECG and telemetry should be performed in cases with high clinical suspicion of Lyme carditis and real-time ambulatory cardiac telemetry may also be particularly useful for such patients [18]. Other tests like echocardiography and chest radiography are useful for evaluation of heart size, heart function, and the presence of pericardial effusion and pulmonary congestion [5], [9], [12]. Most cases of Lyme carditis exhibit structural and functional cardiac abnormalities that are mild and transient. In patients with AV block, diastolic mitral and tricuspid regurgitation due to dissociation of atrial and ventricular systole are often detected [19], [20] as in this case. Current guidelines do not recommend routine endomyocardial biopsy for diagnosis given the potential focality of myocarditis and the high risk of the procedure [5], although it can be considered in specific cases [21].

## Pathogen and vector factors

Analysis of autopsy tissue samples support the disease mechanism of spirochete cardiac tropism during early disease dissemination [22] and inflammatory response [13], [22]. *Borrelia*’s cardiac tropism appears related to expression of surface proteins including P66 and decorin binding proteins [22], [23]. Variation in this protein expression may explain differences in cardiac tropism among different *Borrelia* species [22], [23]. *B. burgdorferi* spirochetes adhere to the extracellular matrix during disseminated infection by binding decorin through specific decorin binding proteins (Dbp), particularly Dbp A [2], [24], [25], [26], [27]. Murine models involving decorin binding protein A (Dbp-A) knock-out mice have shown this protein plays a central role in heart infection, being necessary for cardiac localization of spirochetes [28] while, conversely, cardiac infection is diminished in decorin knock-out mice [29]. It is possible that cardiac-specific modifications of decorin’s glycosaminoglycan groups may alter *B. burgdorferi* spirochete adhesion [22].

Furthermore, the metabolism of *B. burgdorferi* helps to explain the occurrence of conduction defects. Unlike cardiomyocytes, which utilize fat oxidation for energy production, the myocytes of the AV node contain glycogen granules that are broken down into glucose; Spirochetes, including *B. burgdorferi*, have a predilection for glucose as an energy source which may explain the conduction abnormalities in Lyme carditis [30], [31].

These molecular differences in *B. burgdorferi* may explain geographic variation in disease. For example, cardiac involvement has an estimated incidence, if untreated, of 0.3–4% in Europe and 4–10% in the US [5]. Further differences in host tick species (*I. scapularis* and *I. pacificus* in eastern and western North America, *I. ricinus* in Europe) and the main reservoirs of *B. burgdorferi* may further contribute to different epidemiology of Lyme disease globally [4].

## Host factors and disease risk

Most tissue damage in Lyme carditis seems to result from host inflammatory reactions related to both innate and adaptive immune responses [2], [5]. During the early infection phase, patients' mononuclear cells have heightened responsiveness to *B. burgdorferi* antigens and mitogens, as well as decreased suppressor-cell and natural-killer-cell activity [8]. Cryoprecipitates, circulating immune complexes, and elevated total serum IgM levels can be found, leading to the activation of the classical complement pathway early in pathogenesis, while specific IgG antibodies develop gradually [8]. Some degree of vascular damage occurs due to spirochetes or immune complexes in and around blood vessels. All affected tissues show lymphocytic infiltration with plentiful plasma cells [8].

Younger patients demonstrate a more robust and diversified immune response to Lyme infection [32] that can lead to development of Systemic Inflammatory Response Syndrome (SIRS), an exaggerated defense response of the body that involves immunological alterations and acute-phase reactant release. This may explain why young people are more likely to experience serious cardiac involvement. Interestingly, while there is no gender difference in the prevalence of Lyme disease overall [6], [9], [10], [12], there is a marked 3:1 male-to-female predominance of complete heart block. This may be related to sex differences in immune response, together with different behaviors associated with tick exposure but this needs further investigation.

## Management

The cornerstone of management of Lyme carditis is supportive care and antimicrobial therapy [5], [33]. Heart block due to Lyme carditis is mostly reversible [5], [11], [33] and correct diagnosis avoids permanent pacemaker placement and its long-term complications. Adults with mild Lyme carditis, presenting only with first-degree AVB, with PR < 300 ms, can be treated with outpatient oral antibacterials, generally doxycycline 100 mg oral twice daily for 14–21 days. Inpatient care is recommended for patients with severe AV block with a PR interval > 300 ms, second or third degree AVB, in whom continuous ECG monitoring is recommended. In these patients, antibacterial therapy with IV ceftriaxone 2 g once daily is suggested, with conversion to oral therapy upon evidence of clinical improvement. [33].

It has been reported that 35–59% of patients with high-grade AV block, (secondary degree type 2 or type 3 [complete] heart block) undergo temporary pacing, either transcutaneous or transvenous [5], [10]. More than 90% of patients with high grade heart block show resolution within one week of beginning antibacterial therapy, therefore temporary rather than permanent pacemaker placement is preferred [5], [11], [33]. To further evaluate patients in whom temporary pacing was required and for whom restoration of 1:1 AV conduction occurs, stress ECG has been suggested to assess AV conduction stability at higher heart rates [14], [33]. Although there are case reports demonstrating the need for permanent pacing in Lyme carditis despite correct antibacterial therapy, this is rare [5].

## Conclusion

Timely diagnosis and treatment of heart block due to Lyme carditis can lead to immediate and life-saving temporary pacing during initiation of antibiotic therapy, while avoiding unnecessary permanent pacemaker placement. Clinical suspicion for Lyme carditis should be high for young patients with unexplained high grade heart block, particularly in Lyme disease endemic areas. The pathogenesis of this condition is not well understood but it is likely to result from a complex interplay of host, pathogen and disease related factors.

## Disclosures

This research did not receive any specific grant from funding agencies in the public, commercial, or not-for-profit sectors.

## Ethical approval

N/A.

## Consent

Patient signed a standard Mayo Clinic research authorization.

## Funding

None.

## CRediT authorship contribution statement

Maria Chiara Carnazzo – Drafting and revision of the article, Celine Scholin – Writing – Drafting and revision of the article, Fnu Shweta, MBBS – Writing – Drafting and revision of the article, Andrew Calvin, MD, MPH – Conception, acquisition and analysis of data, drafting and revision of the article.

## Declaration of Competing Interest

None.
